# (*S*)-2-(1-Methyl­indolin-2-yl)-1,3-diphenyl­propan-2-ol

**DOI:** 10.1107/S1600536811019143

**Published:** 2011-05-25

**Authors:** Ning Lin, Miao-Miao Chen, Yan-Qiu Deng, Ren-Shi Luo, Seik Weng Ng

**Affiliations:** a Institute of Drug Synthesis and Pharmaceutical Processes, School of Pharmaceutical Sciences, Sun Yat-sen University, Guangzhou 510006, People’s Republic of China; b Department of Chemistry, University of Malaya, 50603 Kuala Lumpur, Malaysia

## Abstract

The five-membered ring in the the title mol­ecule, C_24_H_25_NO, fused with the phenyl­ene ring, is almost planar (r.m.s. deviation = 0.023 Å), with the methyl­ene C atom deviating most from this mean plane [0.031 (1) Å]. The tertiary N atom shows a flattened pyramidal configuration [Σ(angles at N) = 350.3 (6)°].

## Related literature

For the synthesis of the methyl (*S*)-1-methyl­indoline-2-carboxyl­ate reactant, see: Torisu *et al.* (2003 ▶).
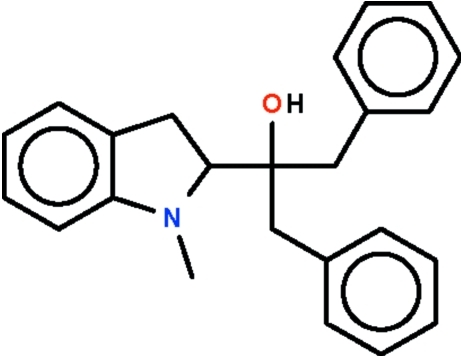


## Experimental

### 

#### Crystal data

C_24_H_25_NO
*M* *_r_* = 343.45
Orthorhombic, 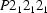

*a* = 9.8501 (6) Å
*b* = 12.0880 (7) Å
*c* = 15.4883 (10) Å
*V* = 1844.2 (2) Å^3^
*Z* = 4
Mo *K*α radiation
μ = 0.08 mm^−1^
*T* = 293 K
0.40 × 0.20 × 0.20 mm

#### Data collection

Bruker SMART CCD diffractometer
10040 measured reflections
2341 independent reflections
2118 reflections with *I* > 2σ(*I*)
*R*_int_ = 0.052

#### Refinement

*R*[*F* ^2^ > 2σ(*F* ^2^)] = 0.044
*wR*(*F* ^2^) = 0.120
*S* = 1.05
2341 reflections
240 parameters
1 restraint
H atoms treated by a mixture of independent and constrained refinement
Δρ_max_ = 0.25 e Å^−3^
Δρ_min_ = −0.18 e Å^−3^

### 

Data collection: *SMART* (Bruker, 2001 ▶); cell refinement: *SAINT* (Bruker, 2001 ▶); data reduction: *SAINT*; program(s) used to solve structure: *SHELXS97* (Sheldrick, 2008 ▶); program(s) used to refine structure: *SHELXL97* (Sheldrick, 2008 ▶); molecular graphics: *X-SEED* (Barbour, 2001 ▶); software used to prepare material for publication: *publCIF* (Westrip, 2010 ▶).

## Supplementary Material

Crystal structure: contains datablocks global, I. DOI: 10.1107/S1600536811019143/zs2115sup1.cif

Structure factors: contains datablocks I. DOI: 10.1107/S1600536811019143/zs2115Isup2.hkl

Supplementary material file. DOI: 10.1107/S1600536811019143/zs2115Isup3.cml

Additional supplementary materials:  crystallographic information; 3D view; checkCIF report

## Figures and Tables

**Fig. 1.**
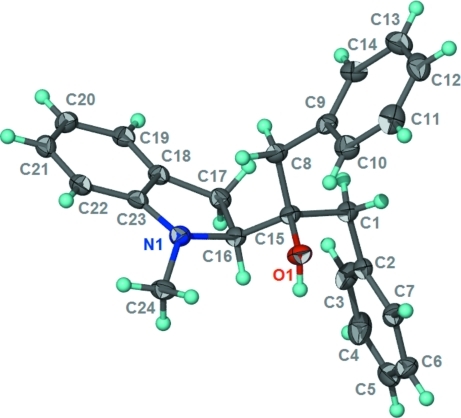
Thermal ellipsoid plot (Barbour, 2001) of C24H25NO at the 50% probability level. Hydrogen atoms are drawn as spheres of arbitrary radius.

## supplementary crystallographic information

### Comment

The optically active compound (Scheme I) is intended for a study on its pharmaceutical property in expectation of activity more enhanced than that of its precursor, methyl (*S*)-1-methylindoline-2-carboxylate (Torisu *et al.*, 2003), which possesses the crucial indoline unit. The five-membered ring in the C_24_H_25_NO molecule is almost planar (r.m.s. deviation 0.023 Å), with the methylene C atom deviating most from this mean plane by 0.031 (1) Å (Fig. 1). The tertiary N atom shows a flattened pyramidal configuration [Σ_angles at N_ 350.3 (6) °]. The hydroxy group is not involved in a hydrogen-bonding interaction.

### Experimental

The Grignard reagent, benzyl magnesium bromide (40 ml, 50 mmol; 1.25 *M* in ether), was added dropwise at 273 K to a solution of methyl (*S*)-1-methylindoline-2-carboxylate (Torisu *et al.*, 2003) (2.39 g, 12.5 mmol) dissolved in ether (20 ml). The mixture was warmed to room temperature and stirring was continued for 24 h under a nitrogen atmosphere. The reaction was quenched with saturated ammonium chloride (20 ml). The organic compound was extracted by using dichloromethane (3 × 15 ml). The organic extract was dried over magnesium sulfate. The solvent was removed and the product separated by column chromatography with hexane/ethyl acetate (45:1) as eluant to afford (*S*)-2-(1-methylindolin-2-yl)-1,3-diphenylpropan-2-ol (3.95 g) in nearly quantative yield.

### Refinement

Carbon-bound H-atoms were placed in calculated positions (C—H = 0.93–0.97 Å) and were included in the refinement in the riding model approximation, with *U*(H) set to 1.2–1.5*U*_eq_(C). 1681 Friedel pairs were merged. The hydroxy H-atom was located in a difference Fourier map, and was refined with a distance restraint of O–H = 0.84±0.01 Å; its displacement parameter was refined. The hydroxy group is not engaged in any hydrogen bonding. The absolute configuration was assumed to be that of the reactant.

### Crystal data

**Table:**

C_24_H_25_NO	*F*(000) = 736
*M**_r_* = 343.45	*D*_x_ = 1.237 Mg m^−^^3^
Orthorhombic, *P*2_1_2_1_2_1_	Mo *K*α radiation, λ = 0.71073 Å
Hall symbol: P 2ac 2ab	Cell parameters from 3877 reflections
*a* = 9.8501 (6) Å	θ = 2.5–27.1°
*b* = 12.0880 (7) Å	µ = 0.08 mm^−^^1^
*c* = 15.4883 (10) Å	*T* = 293 K
*V* = 1844.2 (2) Å^3^	Block, colorless
*Z* = 4	0.40 × 0.20 × 0.20 mm

### Data collection

**Table:**

Bruker SMART CCD diffractometer	2118 reflections with *I* > 2σ(*I*)
Radiation source: fine-focus sealed tube	*R*_int_ = 0.052
graphite	θ_max_ = 27.5°, θ_min_ = 2.1°
φ and ω scans	*h* = −12→9
10040 measured reflections	*k* = −15→15
2341 independent reflections	*l* = −20→18

### Refinement

**Table:**

Refinement on *F*^2^	Primary atom site location: structure-invariant direct methods
Least-squares matrix: full	Secondary atom site location: difference Fourier map
*R*[*F*^2^ > 2σ(*F*^2^)] = 0.044	Hydrogen site location: inferred from neighbouring sites
*wR*(*F*^2^) = 0.120	H atoms treated by a mixture of independent and constrained refinement
*S* = 1.05	*w* = 1/[σ^2^(*F*_o_^2^) + (0.0715*P*)^2^ + 0.2207*P*] where *P* = (*F*_o_^2^ + 2*F*_c_^2^)/3
2341 reflections	(Δ/σ)_max_ = 0.001
240 parameters	Δρ_max_ = 0.25 e Å^−^^3^
1 restraint	Δρ_min_ = −0.18 e Å^−^^3^

### Fractional atomic coordinates and isotropic or equivalent isotropic displacement parameters (Å^2^)

**Table:**

	*x*	*y*	*z*	*U*_iso_*/*U*_eq_	
O1	0.15461 (16)	0.34594 (13)	0.80221 (11)	0.0323 (4)	
H1	0.174 (3)	0.357 (2)	0.7499 (9)	0.045 (8)*	
N1	0.01846 (18)	0.55982 (17)	0.80990 (12)	0.0308 (4)	
C1	0.3692 (2)	0.42422 (18)	0.85258 (15)	0.0297 (5)	
H1A	0.4052	0.4780	0.8930	0.036*	
H1B	0.3908	0.3513	0.8747	0.036*	
C2	0.4405 (2)	0.43916 (18)	0.76668 (15)	0.0285 (5)	
C3	0.4989 (3)	0.5395 (2)	0.74430 (19)	0.0399 (6)	
H3	0.4962	0.5979	0.7833	0.048*	
C4	0.5614 (3)	0.5546 (2)	0.6646 (2)	0.0497 (7)	
H4	0.5993	0.6227	0.6505	0.060*	
C5	0.5668 (2)	0.4677 (2)	0.60614 (18)	0.0442 (7)	
H5	0.6072	0.4776	0.5524	0.053*	
C6	0.5125 (2)	0.3670 (2)	0.62797 (17)	0.0410 (6)	
H6	0.5174	0.3083	0.5893	0.049*	
C7	0.4499 (2)	0.3523 (2)	0.70783 (17)	0.0336 (5)	
H7	0.4139	0.2835	0.7220	0.040*	
C8	0.1523 (2)	0.42783 (19)	0.94128 (14)	0.0305 (5)	
H8A	0.1791	0.4931	0.9734	0.037*	
H8B	0.0541	0.4292	0.9364	0.037*	
C9	0.1919 (2)	0.32691 (18)	0.99352 (15)	0.0289 (5)	
C10	0.1463 (2)	0.22140 (19)	0.97106 (17)	0.0365 (5)	
H10	0.0929	0.2124	0.9220	0.044*	
C11	0.1792 (3)	0.1302 (2)	1.0205 (2)	0.0441 (6)	
H11	0.1472	0.0607	1.0046	0.053*	
C12	0.2595 (3)	0.1412 (2)	1.09372 (18)	0.0428 (6)	
H12	0.2821	0.0795	1.1266	0.051*	
C13	0.3054 (3)	0.2453 (2)	1.11701 (18)	0.0434 (6)	
H13	0.3587	0.2540	1.1661	0.052*	
C14	0.2717 (2)	0.3366 (2)	1.06703 (15)	0.0361 (5)	
H14	0.3034	0.4060	1.0832	0.043*	
C15	0.2125 (2)	0.43663 (17)	0.84985 (14)	0.0275 (4)	
C16	0.1685 (2)	0.54640 (17)	0.80736 (15)	0.0282 (5)	
H16	0.1975	0.5455	0.7468	0.034*	
C17	0.2251 (2)	0.65260 (17)	0.85054 (15)	0.0302 (5)	
H17A	0.2844	0.6342	0.8984	0.036*	
H17B	0.2752	0.6970	0.8092	0.036*	
C18	0.1009 (2)	0.71306 (17)	0.88161 (14)	0.0271 (4)	
C19	0.0897 (2)	0.81040 (18)	0.92767 (15)	0.0297 (5)	
H19	0.1671	0.8479	0.9459	0.036*	
C20	−0.0393 (2)	0.85191 (19)	0.94665 (15)	0.0325 (5)	
H20	−0.0485	0.9176	0.9773	0.039*	
C21	−0.1534 (2)	0.79467 (19)	0.91952 (15)	0.0327 (5)	
H21	−0.2389	0.8228	0.9327	0.039*	
C22	−0.1440 (2)	0.69653 (18)	0.87326 (14)	0.0305 (5)	
H22	−0.2216	0.6588	0.8558	0.037*	
C23	−0.0148 (2)	0.65606 (17)	0.85368 (14)	0.0268 (4)	
C24	−0.0615 (2)	0.5207 (2)	0.73715 (16)	0.0342 (5)	
H24A	−0.1539	0.5446	0.7438	0.051*	
H24B	−0.0584	0.4414	0.7351	0.051*	
H24C	−0.0250	0.5504	0.6846	0.051*	

### Atomic displacement parameters (Å^2^)

**Table:**

	*U*^11^	*U*^22^	*U*^33^	*U*^12^	*U*^13^	*U*^23^
O1	0.0333 (8)	0.0377 (8)	0.0259 (8)	−0.0021 (7)	0.0010 (7)	−0.0070 (7)
N1	0.0245 (8)	0.0407 (10)	0.0271 (9)	0.0026 (8)	−0.0034 (7)	−0.0052 (8)
C1	0.0298 (10)	0.0330 (10)	0.0263 (11)	0.0031 (8)	−0.0014 (9)	−0.0031 (10)
C2	0.0235 (9)	0.0340 (10)	0.0280 (11)	0.0048 (8)	−0.0020 (8)	−0.0022 (9)
C3	0.0355 (10)	0.0367 (12)	0.0476 (15)	0.0037 (10)	0.0114 (11)	−0.0043 (11)
C4	0.0403 (13)	0.0460 (14)	0.0627 (19)	0.0086 (12)	0.0166 (13)	0.0142 (14)
C5	0.0285 (11)	0.0723 (18)	0.0318 (13)	0.0137 (11)	0.0061 (10)	0.0137 (13)
C6	0.0280 (10)	0.0625 (15)	0.0326 (13)	0.0086 (10)	−0.0017 (10)	−0.0152 (12)
C7	0.0263 (10)	0.0386 (11)	0.0359 (12)	0.0026 (9)	−0.0010 (9)	−0.0068 (11)
C8	0.0311 (10)	0.0358 (11)	0.0245 (11)	0.0026 (9)	0.0024 (9)	−0.0050 (9)
C9	0.0244 (9)	0.0364 (11)	0.0259 (10)	0.0009 (8)	0.0058 (8)	−0.0022 (9)
C10	0.0313 (10)	0.0413 (12)	0.0368 (14)	−0.0032 (10)	−0.0024 (10)	−0.0014 (11)
C11	0.0409 (13)	0.0373 (11)	0.0540 (17)	−0.0025 (10)	0.0047 (13)	0.0012 (12)
C12	0.0383 (12)	0.0454 (13)	0.0446 (14)	0.0063 (10)	0.0054 (11)	0.0133 (13)
C13	0.0414 (12)	0.0582 (15)	0.0306 (13)	0.0023 (11)	−0.0020 (11)	0.0073 (12)
C14	0.0396 (11)	0.0421 (13)	0.0265 (11)	−0.0045 (10)	0.0006 (10)	−0.0010 (10)
C15	0.0265 (9)	0.0326 (10)	0.0233 (10)	0.0027 (9)	−0.0008 (8)	−0.0050 (9)
C16	0.0255 (10)	0.0353 (11)	0.0239 (10)	0.0020 (9)	−0.0006 (9)	−0.0020 (9)
C17	0.0272 (10)	0.0344 (10)	0.0288 (11)	0.0002 (9)	−0.0034 (9)	−0.0003 (10)
C18	0.0272 (9)	0.0309 (10)	0.0231 (10)	0.0000 (8)	0.0000 (9)	0.0046 (8)
C19	0.0308 (10)	0.0331 (10)	0.0251 (11)	−0.0014 (9)	−0.0002 (9)	0.0028 (9)
C20	0.0395 (11)	0.0308 (10)	0.0271 (11)	0.0038 (9)	0.0050 (10)	0.0003 (9)
C21	0.0286 (10)	0.0404 (11)	0.0290 (12)	0.0072 (9)	0.0033 (9)	0.0065 (10)
C22	0.0270 (9)	0.0392 (11)	0.0254 (11)	−0.0001 (9)	−0.0017 (9)	0.0054 (9)
C23	0.0297 (10)	0.0312 (10)	0.0195 (10)	0.0010 (8)	−0.0019 (8)	0.0046 (9)
C24	0.0310 (10)	0.0458 (12)	0.0258 (11)	−0.0029 (9)	−0.0044 (9)	−0.0040 (10)

### Geometric parameters (Å, °)

**Table:**

O1—C15	1.439 (3)	C10—H10	0.9300
O1—H1	0.843 (10)	C11—C12	1.389 (4)
N1—C23	1.386 (3)	C11—H11	0.9300
N1—C24	1.454 (3)	C12—C13	1.385 (4)
N1—C16	1.487 (3)	C12—H12	0.9300
C1—C2	1.516 (3)	C13—C14	1.388 (4)
C1—C15	1.551 (3)	C13—H13	0.9300
C1—H1A	0.9700	C14—H14	0.9300
C1—H1B	0.9700	C15—C16	1.543 (3)
C2—C3	1.386 (3)	C16—C17	1.551 (3)
C2—C7	1.394 (3)	C16—H16	0.9800
C3—C4	1.391 (4)	C17—C18	1.504 (3)
C3—H3	0.9300	C17—H17A	0.9700
C4—C5	1.388 (4)	C17—H17B	0.9700
C4—H4	0.9300	C18—C19	1.380 (3)
C5—C6	1.371 (4)	C18—C23	1.400 (3)
C5—H5	0.9300	C19—C20	1.397 (3)
C6—C7	1.393 (4)	C19—H19	0.9300
C6—H6	0.9300	C20—C21	1.385 (3)
C7—H7	0.9300	C20—H20	0.9300
C8—C9	1.515 (3)	C21—C22	1.389 (3)
C8—C15	1.539 (3)	C21—H21	0.9300
C8—H8A	0.9700	C22—C23	1.397 (3)
C8—H8B	0.9700	C22—H22	0.9300
C9—C14	1.389 (3)	C24—H24A	0.9600
C9—C10	1.396 (3)	C24—H24B	0.9600
C10—C11	1.381 (4)	C24—H24C	0.9600
			
C15—O1—H1	106 (2)	C12—C13—H13	120.0
C23—N1—C24	121.61 (18)	C14—C13—H13	120.0
C23—N1—C16	109.84 (17)	C13—C14—C9	121.7 (2)
C24—N1—C16	118.84 (18)	C13—C14—H14	119.1
C2—C1—C15	115.22 (18)	C9—C14—H14	119.1
C2—C1—H1A	108.5	O1—C15—C8	105.45 (17)
C15—C1—H1A	108.5	O1—C15—C16	108.96 (17)
C2—C1—H1B	108.5	C8—C15—C16	110.09 (17)
C15—C1—H1B	108.5	O1—C15—C1	109.54 (17)
H1A—C1—H1B	107.5	C8—C15—C1	110.58 (18)
C3—C2—C7	117.9 (2)	C16—C15—C1	111.99 (18)
C3—C2—C1	121.1 (2)	N1—C16—C15	111.21 (18)
C7—C2—C1	121.0 (2)	N1—C16—C17	104.81 (17)
C2—C3—C4	121.3 (2)	C15—C16—C17	115.25 (17)
C2—C3—H3	119.3	N1—C16—H16	108.4
C4—C3—H3	119.3	C15—C16—H16	108.4
C5—C4—C3	119.8 (3)	C17—C16—H16	108.4
C5—C4—H4	120.1	C18—C17—C16	104.33 (17)
C3—C4—H4	120.1	C18—C17—H17A	110.9
C6—C5—C4	119.7 (2)	C16—C17—H17A	110.9
C6—C5—H5	120.2	C18—C17—H17B	110.9
C4—C5—H5	120.2	C16—C17—H17B	110.9
C5—C6—C7	120.3 (2)	H17A—C17—H17B	108.9
C5—C6—H6	119.8	C19—C18—C23	121.0 (2)
C7—C6—H6	119.8	C19—C18—C17	130.1 (2)
C6—C7—C2	120.9 (2)	C23—C18—C17	108.90 (18)
C6—C7—H7	119.6	C18—C19—C20	119.2 (2)
C2—C7—H7	119.6	C18—C19—H19	120.4
C9—C8—C15	116.63 (17)	C20—C19—H19	120.4
C9—C8—H8A	108.1	C21—C20—C19	119.7 (2)
C15—C8—H8A	108.1	C21—C20—H20	120.2
C9—C8—H8B	108.1	C19—C20—H20	120.2
C15—C8—H8B	108.1	C20—C21—C22	121.9 (2)
H8A—C8—H8B	107.3	C20—C21—H21	119.0
C14—C9—C10	117.6 (2)	C22—C21—H21	119.0
C14—C9—C8	121.1 (2)	C21—C22—C23	118.2 (2)
C10—C9—C8	121.3 (2)	C21—C22—H22	120.9
C11—C10—C9	121.1 (2)	C23—C22—H22	120.9
C11—C10—H10	119.5	N1—C23—C22	127.99 (19)
C9—C10—H10	119.5	N1—C23—C18	111.84 (18)
C10—C11—C12	120.6 (2)	C22—C23—C18	120.14 (19)
C10—C11—H11	119.7	N1—C24—H24A	109.5
C12—C11—H11	119.7	N1—C24—H24B	109.5
C13—C12—C11	119.0 (2)	H24A—C24—H24B	109.5
C13—C12—H12	120.5	N1—C24—H24C	109.5
C11—C12—H12	120.5	H24A—C24—H24C	109.5
C12—C13—C14	119.9 (2)	H24B—C24—H24C	109.5
			
C15—C1—C2—C3	97.5 (3)	C23—N1—C16—C17	−3.8 (2)
C15—C1—C2—C7	−82.7 (2)	C24—N1—C16—C17	142.8 (2)
C7—C2—C3—C4	1.9 (4)	O1—C15—C16—N1	61.9 (2)
C1—C2—C3—C4	−178.3 (2)	C8—C15—C16—N1	−53.2 (2)
C2—C3—C4—C5	−0.5 (4)	C1—C15—C16—N1	−176.71 (18)
C3—C4—C5—C6	−1.0 (4)	O1—C15—C16—C17	−178.96 (18)
C4—C5—C6—C7	1.1 (4)	C8—C15—C16—C17	65.9 (2)
C5—C6—C7—C2	0.4 (3)	C1—C15—C16—C17	−57.6 (2)
C3—C2—C7—C6	−1.9 (3)	N1—C16—C17—C18	5.1 (2)
C1—C2—C7—C6	178.3 (2)	C15—C16—C17—C18	−117.5 (2)
C15—C8—C9—C14	113.0 (2)	C16—C17—C18—C19	177.1 (2)
C15—C8—C9—C10	−68.9 (3)	C16—C17—C18—C23	−4.9 (2)
C14—C9—C10—C11	0.3 (3)	C23—C18—C19—C20	0.2 (3)
C8—C9—C10—C11	−177.9 (2)	C17—C18—C19—C20	178.0 (2)
C9—C10—C11—C12	−0.5 (4)	C18—C19—C20—C21	0.4 (3)
C10—C11—C12—C13	0.6 (4)	C19—C20—C21—C22	−0.3 (3)
C11—C12—C13—C14	−0.5 (4)	C20—C21—C22—C23	−0.4 (3)
C12—C13—C14—C9	0.3 (4)	C24—N1—C23—C22	37.3 (3)
C10—C9—C14—C13	−0.2 (3)	C16—N1—C23—C22	−177.2 (2)
C8—C9—C14—C13	178.0 (2)	C24—N1—C23—C18	−144.7 (2)
C9—C8—C15—O1	65.5 (2)	C16—N1—C23—C18	0.8 (3)
C9—C8—C15—C16	−177.09 (18)	C21—C22—C23—N1	178.8 (2)
C9—C8—C15—C1	−52.8 (2)	C21—C22—C23—C18	1.0 (3)
C2—C1—C15—O1	68.1 (2)	C19—C18—C23—N1	−179.06 (19)
C2—C1—C15—C8	−176.10 (17)	C17—C18—C23—N1	2.7 (2)
C2—C1—C15—C16	−52.9 (2)	C19—C18—C23—C22	−0.9 (3)
C23—N1—C16—C15	121.39 (19)	C17—C18—C23—C22	−179.1 (2)
C24—N1—C16—C15	−92.0 (2)		
